# Early intervention to prevent adverse child emotional and behavioural development following maternal depression in pregnancy: study protocol for a randomised controlled trial

**DOI:** 10.1186/s40359-023-01244-w

**Published:** 2023-08-04

**Authors:** Jeannette Milgrom, Yafit Hirshler, Charlene Holt, Helen Skouteris, Megan Galbally, Christine East, Vivette Glover, John Reece, Kieran J. O’Donnell, Susan P. Walker, Shannon Malloy, Alan W. Gemmill

**Affiliations:** 1 Parent-Infant Research Institute, Austin Health, 300 Waterdale Road, Heidelberg Heights, VIC 3081 Australia; 2 Melbourne School of Psychological Sciences, University of Melbourne, Grattan Street, Parkville, VIC 3010 Australia; 3 Health and Social Care Unit, School of Public Health and Preventive Medicine, Monash University, 553 St Kilda Road, Melbourne, VIC 3004 Australia; 4 Health Futures Institute, Murdoch University, 90 South Street, Murdoch, WA 6150 Australia; 5 School of Clinical Sciences, Monash University, Clayton, VIC 3168 Australia; 6 Mental Health, Program Monash Medical Centre, Monash Health, 246 Clayton Road, Clayton, VIC 3168 Australia; 7 Judith Lumley Centre, School of Nursing and Midwifery, La Trobe University, Plenty Rd & Kingsbury Drive, Bundoora, VIC 3086 Australia; 8 Mercy Hospital for Women, 163 Studley Road, Heidelberg, VIC 3084 Australia; 9 Institute of Reproductive and Developmental Biology, Imperial College London, Du Cane Road, London, W12 ONN UK; 10 School of Psychological Sciences, Australian College of Applied Professions, 123 Lonsdale Street, Melbourne, VIC 3000 Australia; 11 Yale Child Study Center, Yale School of Medicine, 230 South Frontage Road, New Haven, CT 06519 USA; 12 Department of Obstetrics Gynecology and Reproductive Sciences, 230 South Frontage Road, New Haven, CT 06519 USA; 13 Department of Psychiatry, McGill University, 1033 Pine Avenue West, Montreal, QC H3A 1A1 Canada; 14 Melbourne Medical School, University of Melbourne, Grattan Street, Parkville, VIC 3010 Australia; 15 Ovia Health, 263 Summer Street, Boston, MA 02210 USA

**Keywords:** Antenatal depression, Antenatal anxiety, Child emotional and behavioural development, Child internalising behaviour, Cognitive behavioural therapy, Psychological treatment

## Abstract

**Background:**

Substantial evidence indicates that maternal depression during pregnancy (i.e., antenatal depression) is associated not only with maternal wellbeing but also with child emotional and behavioural development. Children of antenatally depressed women are at risk of emotional and behavioural problems, including internalising problems (e.g., anxiety and depression) and externalising problems (e.g., attention problems), that may last at least to adolescence. These enduring effects also constitute an enormous economic cost. Despite the seriousness of this problem, until recently there existed very few controlled studies evaluating whether active psychological treatment for antenatal depression can prevent adverse child outcomes. Our previous pilot randomised controlled trial (RCT) exploring the effect of cognitive behavioural therapy (CBT) for antenatal depression on child outcomes showed promising results. We aim to assess whether treating antenatal depression with an evidence-based 8-week structured CBT program can prevent or ameliorate adverse child developmental outcomes at 2 years of age.

**Methods:**

Pregnant women ≤ 30 weeks gestation diagnosed with a depressive disorder are recruited and randomised to CBT or treatment as usual (TAU). The target sample size is 230 and the primary outcome measure is the infant Internalising scale of the Child Behaviour Checklist (CBCL) at 24 months of age. Secondary infant outcome measures at 24 months are the Externalising scale of the CBCL and the motor and cognitive development subscales of the Ages & Stages Questionnaire (ASQ-3). Additional secondary outcome measures are subscales of the Revised Infant Behaviour Questionnaire (IBQ-R), ASQ-3 and the ASQ-Socio-Emotional (ASQ-SE) at 3 and 12 months of age and the quality of mother-infant interaction at 3 and 24 months. Maternal measures, including demographic data, depression diagnosis, depressive and anxiety symptoms, perceived stress and parenting stress, are collected across all time points.

**Discussion:**

The trial is ongoing and recruitment was slowed due to the COVID-19 pandemic. If results suggest a beneficial effect of antenatal depression treatment on infant outcomes, the project could have repercussions for standard antenatal care, for maternal and infant health services and for preventing the intergenerational transmission of mental health disorders.

**Trial registration:**

Australia and New Zealand Clinical Trials Register: ACTRN12618001925235 Date Registered: 27 November 2018.

**Supplementary Information:**

The online version contains supplementary material available at 10.1186/s40359-023-01244-w.

## Background

Depression in pregnancy is an under-recognised clinical condition [1]. In Australia, about 300,000 women give birth per year [2]. Approximately 20% of pregnant women experience a depressive disorder [3], so that over 60,000 families are affected annually. Depression has profound repercussions for maternal wellbeing, as well as infant development, and imposes one of the highest burdens of any non-communicable disease in Australia and worldwide [4]. Failure to treat perinatal depression and associated anxiety is common and incurs enormous economic costs. The London School of Economics estimates the cost to society of perinatal mental illness at £8.1 billion for every one-year UK cohort of births, overwhelmingly attributable to the enduring impact of maternal mental illness on children [5]. An equivalent estimate for Australia exceeds $7 billion for each one-year cohort [6].

### The link between antenatal depression and adverse child emotional and behavioural development

There exists a body of knowledge, which taken as a whole, is highly suggestive of an association between antenatal maternal depression and internalising and externalising problems in the offspring throughout infancy and adolescence [7, 8]. Rogers and colleagues [9] reported in their meta-analysis (n = 35 prospective studies) small-to-moderate associations (some of which are indicated as clinically important) between antenatal depression and various child measures, including socio-emotional development as a composite, internalising behaviours and externalising behaviours. Similarly, the meta-analysis by Madigan and colleagues [10] reported a strong association between antenatal depression and socio-emotional development (n = 50 prospective studies) and with internalising and externalising problems (n = 20 studies). According to the meta-analysis by Tirumalaraju and colleagues ([11]; n = 4 prospe ctive studies), the association between antenatal depression and offspring depression is also evident in adulthood.

A number of studies, some of which included in the above meta-analyses, demonstrated strong associations between antenatal depression and offspring emotional and behavioural problems, even after controlling for postnatal confounders, such as maternal postnatal depression. For example, the associations between antenatal depressive symptoms and internalising and externalising problems in 1.9- to 5.9-year-old children [12] or behavioural and emotional problems in 4- to 13-year-old children [13] remained significant after controlling for various confounders, including postnatal depressive symptoms. Likewise, in adolescent and adult offspring, the risk of anxiety diagnosis [14] and depression diagnosis [15, 16] remained heightened after adjusting for maternal postnatal depression. These results suggest that the association between antenatal depression and offspring behavioural problems is at least partly independent of postnatal depression, therefore implying these difficulties originate in utero and arise via fetal programming mechanisms [17, 18]. This is further supported by the notion that the relationships between antenatal depression and behavioural and emotional difficulties were persistent across childhood [13].

Given that most existing evidence from human studies is correlational, an experimental design by intervening antenatally is needed to demonstrate causality [8, 19]. A demonstration that treating maternal depression in pregnancy ameliorates negative child outcomes would be consistent with a causal link. To our knowledge, only a few RCTs, including our pilot studies, have evaluated the effect of treating antenatal depression with structured psychological approaches (e.g., CBT and interpersonal psychotherapy) on child behavioural outcomes [20–23], but other studies are underway (e.g., [7]). Whilst these studies have shown some promising results [22, 23], a larger sample size is needed to detect a minimum clinically important difference (MCID) in internalising behaviour at 24 months, the specific primary outcome of this study.

Support for the causal link between antenatal depression and adverse child outcomes also emerges from animal prenatal stress models where genetics and postnatal environment can be controlled [24, 25]. At the same time, human studies exploring functional and structural changes in the brains of neonates exposed to antenatal depression are notable as they reduce the confounding effect of postnatal influences by targeting early-emerging outcomes [26]. For example, neonates exposed to antenatal depression had greater inverse connectivity between the left amygdala and the dorsal prefrontal cortex (two brain regions implicated in emotional processing and regulation) compared to non-exposed neonates. This amygdala–PFC connectivity was found to mediate the positive relationship between antenatal depression and fetal heart rate reactivity [27]. Variability in neonatal amygdala functional connectivity with the prefrontal cortex in turn predicted internalising behaviour at two years of age [28].

If our research hypothesis that treating maternal depression in pregnancy can prevent adverse child outcomes is supported, this would be consistent with the concept that child outcomes are influenced by fetal programming mechanisms.

### Our CBT intervention for antenatal depression and its effect on child development

Our antenatal CBT treatment for depression, Beating the Blues Before Birth, has been trialled and validated for its effect on child development in a pilot study [22]. In a feasibility study, the treatment has proved to be highly effective at reducing both depression and associated anxiety in a population of pregnant women diagnosed with depression (80% with major depressive disorder) [22]. The pilot RCT that followed is to our knowledge one of only a very few published trials reporting the impact of antenatal depression treatment on child behavioural outcomes [22]. In this pilot RCT (n = 54), substantial reductions were achieved in maternal depression and anxiety in the intervention group compared to TAU. Depression gains were maintained at 9 months postpartum (Cohen’s d = 0.67, p = .05). The intervention group showed excellent treatment adherence. Of a possible eight sessions, women in the intervention attended an average of 6.30 (SD 2.91); 78% attended six or more sessions, and 63% completed all eight sessions. At 9 months, the largest effects [29] on child outcomes were seen on the Infant Behaviour Questionnaire-Revised (IBQ-R: [30]) including subscales measuring negative affectivity (d = 0.84, p = .03) and stress reactivity (d = 1.08, p = .007). Stress reactivity in infants is a specific area that is impacted negatively by maternal mental health difficulties in pregnancy [31]. Several subscales of the Ages & Stages Questionnaire-3 and Ages & Stages Questionnaire Socio-Emotional (ASQ-3 and ASQ-SE: [32], [33]) also showed large effects favouring the intervention group children at 9 months including self-regulation (d = 0.83, p = .03) and communication (d = 0.81, p = .04). Our follow-up study in this cohort demonstrated differences in child behaviour at 24 months [23]. On the CBCL [34], medium-to-large effect sizes [29] were seen in behaviours comprising the Internalising scale, especially ‘withdrawal’ and ‘anxious/depressed’ behaviours (d = 0.57 and 0.59 respectively) and in emotional self-regulation (d = 0.42). Intervention group children also trended towards lower scores (d = 0.5) on the DSM-oriented Anxiety Problems scale (which maps CBCL items to DSM anxiety diagnoses). Furthermore, five out of seven syndrome subscales showed a trend in favour of the intervention group. Motor and cognitive development (assessed with the Bayley Scales: [35]) showed small-to-medium sized effects in the same direction (d = 0.52 and d = 0.26 respectively). While highly encouraging, our pilot work was based on a relatively small sample and the current protocol describes a fully-powered study capable of reliable statistical detection of a MCID in early child behavioural problems. To our knowledge this is the first adequately-powered study with medium-term follow-up to determine whether effectively treating maternal depression exclusively in pregnancy has a beneficial effect on child outcomes.

The intervention study presented here is an RCT comparing CBT treatment with best practice care offered to participants in the TAU group. This highly feasible approach to optimising intergenerational mental health has the potential to provide direct evidence that child emotional and behavioural development and future mental health is protected by timely antenatal depression treatment.

## Methods

### Study aim

This study aims to establish whether adverse child development can be ameliorated by the treatment of depression in pregnant women in an adequately-powered RCT.

The primary outcome will be emotional and behavioural development measured by the Internalising scale of the CBCL at 24 months. The Internalising scale encompasses symptoms that most closely map to the symptoms of affective and anxiety disorders (anxious/depressed symptoms, social withdrawal, emotional reactivity and somatic complaints). There is good evidence that CBCL scores in early childhood, in particular internalising scores, are predictive of later mental health disorders extending into young adulthood [36]; see systematic review [37].

### Study design

**Fig. 1:**
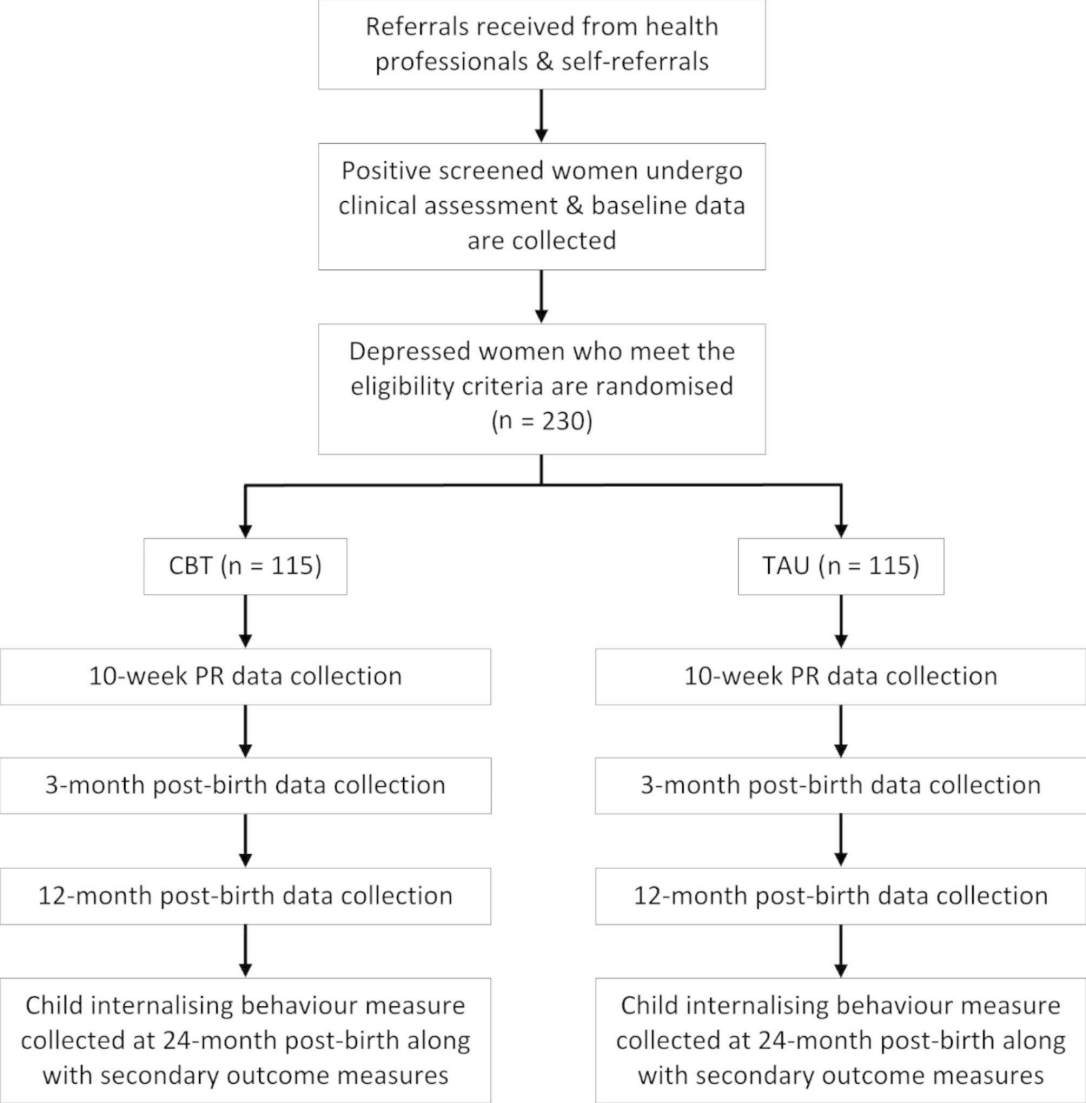
Design of the study PR = post randomisation

This is a multi-centre, parallel, two-group RCT designed to recruit 230 depressed pregnant women with a 1:1 allocation ratio to either CBT or TAU (n = 115 in each condition) within a superiority framework. The design is shown in Fig. 1. This protocol follows the recommendations of Standard Protocol Items: Recommendations for Interventional Trials (SPIRIT: [38]), Guidelines for Reporting Trial Protocols and Completed Trials Modified Due to the COVID-19 Pandemic and Other Extenuating Circumstances (CONSERVE-SPIRIT extension: [39]), and Template for Intervention Description and Replication (TIDieR: [40]). The research is being conducted in line with Consolidated Standards of Reporting Trials (CONSORT) standards [41] and the National Statement on Ethical Conduct in Research Involving Humans.

### Study setting

We are recruiting women across Australia through study advertisement on a free pregnancy tracking mobile application, Ovia Pregnancy. This application supports expectant mothers by providing them with personalised information relevant to their pregnancy. We are also recruiting at major maternity hospitals in Victoria, Australia; Royal Women’s Hospital, Mercy Health, and Monash Health. The advent of COVID-19 slowed the involvement of maternity hospitals until recently. Strategies for achieving adequate participant enrolment to reach target sample size include periodic adjustment of the Ovia study advertisement and meeting regularly with referrers in maternity hospitals including conductance of educational sessions.

### Participants

Participants are adult women who are less than 30 weeks pregnant, diagnosed with a current depressive disorder and gave informed consent to participate in the study.

### Eligibility criteria

Eligibility for the study is assessed in two stages; screening and diagnostic assessment.

#### Screening

Women expressing interest in the study are screened on the basis of the following initial criteria: (1) > 18 of age; (2) ≤ 30 weeks pregnant; (3) not currently receiving treatment for depression or anxiety (medication or psychotherapy); (4) fluent in English; (5) an Edinburgh Postnatal Depression Scale (EPDS: [42]) score equal to or greater than 13, a positive response to one of the Whooley Questions [43], or clinical indication.

Women who meet these initial criteria are referred to the study and are contacted by the research team for an Intake call. Informed consent to participate in the study is obtained electronically by the study co-ordinator following the Intake call.

#### Diagnostic assessment

Following the Intake call, maternal mental health is assessed using the Structured Clinical Interview for the Diagnostic and Statistical Manual of Mental Disorders-5 Clinical version-CV (SCID-5-CV: [44]) to yield a diagnosis of current major depressive episode or depressive episode with insufficient symptoms.

Women with responses reflecting thoughts of self-harm (based on EPDS Item 10 or the SCID) are asked a series of questions to determine intentionality, lethality, access to means, and history of suicide attempts. Women deemed to be at risk requiring crisis or inpatient management are excluded and referred to receive immediate crisis attention. Our protocol follows the principles described in the New South Wales Framework for Suicide Risk Assessment and Management [45].

Women are included in the study if they meet the above-mentioned initial criteria and a DSM-5 diagnosis of major depressive disorder or depressive episode with insufficient symptoms and do not meet one or more of the following exclusion criteria as assessed by the SCID: a) concurrent major psychiatric disorders (particularly psychotic and bipolar disorders; we do not exclude co-morbid anxiety disorders); b) substance use disorder.

### Randomisation

Women diagnosed with a depressive disorder and meeting all other criteria are offered randomisation after all baseline data are collected. Randomisation is completed by the study co-ordinator in a 1:1 ratio to CBT intervention (n = 115) or to TAU (n = 115), using a pre-generated, variable-length, permuted-blocks allocation schedule, stratified by site, with allocation concealment ensured by central, independent administration consistent with CONSORT standards [41]. The study uses secure randomisation services provided by the NHMRC Clinical Trials Centre. Given the nature of the psychological intervention, study participants cannot be blinded beyond the point of treatment allocation. Outcome assessments are conducted by psychologists who are kept blind to treatment allocation, and the primary analysis will also be conducted blind to treatment allocation. Unblinding is permissible when risk is indicated at follow-up and requires referral to a health professional.

### Interventions and comparators

#### Beating the Blues before Birth CBT Intervention

Participants in the CBT intervention group receive seven individual 1-hour weekly sessions and one couple session (if they do not have a partner they can invite a close person such as a family member or a friend). Initially the sessions were delivered in-person and since the advent of COVID-19 via telehealth using a secured platform.

Adapted from our Getting Ahead of Post Natal Depression intervention [46, 47], this antenatal CBT intervention was tailored to address the specific needs of pregnant women concerning issues such as lack of time and physical health with a focus on preparing for transition to parenthood, self-care, and lifestyle. Sessions include psycho-education, activities and discussion on behavioural (Understanding Antenatal Depression & Anxiety, Pleasant Activities, Self-Care & Relaxation in Pregnancy, Assertiveness & Self-esteem) and cognitive strategies (Expectations and Transition to Parenthood, Developing a More Helpful Thinking Style, Challenging My Internal Critic). The couple session provides information and support to partners and includes strategies for effective communication. The final session is focussed on Relapse Prevention. The intervention has proved effective in our previous RCT [22].

Participant booklets and partner booklets, which contain information summarising session content, are provided. The intervention is delivered by psychologists specialising in perinatal mental health and the CBT approach and are trained in the program.

Since 29 November 2018, 366 sessions have been delivered to 51 participants as detailed in the Status section below.

##### Criteria for discontinuing the intervention

Given this is an antenatal intervention it is discontinued when participants give birth prior to the completion of the 8 sessions, in which case a final single postnatal session is delivered prior to the 3-month post-birth time point.

##### Treatment fidelity & Adherence

Psychologists adhere strictly to the detailed protocol and manual ensuring uniform delivery. Following each CBT session, therapists check off the items covered (or re-visited) from the manual and ensure all content is covered as women progress in the treatment. These checklists are closely monitored by the study co-ordinator and to date all session content has been covered apart from exceptional occasions, for example when the participant gave birth unexpectedly. Therapists review the homework tasks completed by participants to ensure participants adhere to therapy.

#### Treatment as usual

Participants in this group are referred to their general practitioner (GP) with the results of their clinical assessment and information on study participation. Women may elect to be referred to their midwife or obstetrician. Health professionals are free to treat or to refer to other services/agencies as they judge appropriate, as would normally happen where specialised programs are not available. We have successfully demonstrated the superiority of our specialised CBT intervention compared to TAU as a control condition in several of our previous randomised trials in this area of work [22, 47, 48].

#### Concomitant care

Although current treatment at the time of enrolment is an exclusion criterion, participation in the study does not preclude women from receiving support at later stages. Thus, women in both groups are free to receive concomitant care as they see appropriate.

### Study outcome measures

Self-reported outcome measures were initially collected via paper questionnaires. With the advent of COVID-19 these are now collected via Qualtrics platform (Qualtrics, Inc., WA, USA). To promote participant retention, we provide $20 reimbursement for completing the questionnaires at each follow-up time point and send SMS reminders for data returns and thank you, birthday, and end of year season’s greetings.

The primary outcome is child emotional and behavioural development measured by the Internalising scale of the CBCL [34] at 24 months of age (corrected age for babies born less than 32 weeks gestation). Secondary outcome measures are assessed when children are 3 months, 12 months, and 24 months of age, and contemporaneous maternal measures are collected across all time points (see Table 1). Earlier developmental measures (3 and 12 months) will allow detection of emerging treatment effects. Earlier follow-up time points will also facilitate imputation of missing or censored end-point data in intention-to-treat analyses. All psychometric instruments are validated, reliable and widely used. Clinician-administered and observer-rated measures are collected blind to treatment allocation.

**Table 1:** Schedule of data collection

	Baseline	10-week post-randomisation	3-month post-birth	12-month post-birth	24-month post-birth
**Infant measures** *Primary Outcome*					
Child Behaviour (Internalising Scale of the CBCL)					**√**
*Secondary Outcomes*					
Child Behaviour (Externalising Scale of the CBCL)					**√**
Observer-rated Child Behaviour (PCERA sub-scales)*			**√**		**√**
Infant Behaviour (IBQ-R)			**√**	**√**	
Infant Developmental Milestones (ASQ-3 and ASQ-SE)			**√**	**√**	**√**
**Maternal measures**					
Maternal Depression Diagnosis (SCID-5-CV)*	**√**		**√**		**√**
Maternal Depression Severity (BDI-II)	**√**	**√**	**√**	**√**	**√**
Maternal Anxiety Severity (BAI)	**√**	**√**	**√**	**√**	**√**
Parenting Stress Index (PSI)			**√**	**√**	**√**
Perceived Stress Scale (PSS)	**√**	**√**	**√**	**√**	**√**
Demographic and Descriptive Data	**√** ^1^		**√** ^1^		

*clinician administered, blinded rating

^1^some of the descriptive questions are included at later time points

#### Primary outcome

Child Behaviour Checklist Internalising Scale (CBCL: [34]). The Internalising scale of the CBCL at 24 months is the primary outcome. The CBCL is one of the most widely used standardised measures in child psychology for evaluating maladaptive behavioural and emotional problems in children 18 months and older. The Internalising scale encompasses symptoms that most closely map to the symptoms of affective and anxiety disorders (anxious/depressed symptoms, social withdrawal, emotional reactivity and somatic complaints). There is good evidence that CBCL scores in early childhood, in particular internalising scores, are predictive of later mental health disorders [36, 37]. Completed by a parent or other caregiver, the CBCL1½-5 contains 99 items, scored 0 = not true, 1 = somewhat true, and 2 = very true or often true, based on the preceding two months, to yield empirically based syndrome scores. The Internalising scale yields a single score calculated as the sum of four syndrome scores (anxious/depressed symptoms, social withdrawal, emotional reactivity, and somatic complaints) and has good-to-excellent internal consistency (alpha = 0.90), inter-rater agreement (r = .75) and test-retest reliability (r = .85).

#### Secondary outcomes

Child Behaviour Checklist Externalising Scale (CBCL: [34]). The Externalising scale comprises syndrome scores for behaviours related to attention and aggression.

Parent-Child Early Relational Assessment (PCERA: [49]), a clinician-rated measure, is based on observations of mother-child behaviours that provides an independent rating of infant behaviour. Videos of a structured task and free play are coded by a trained, blinded observer. All subscale scores demonstrated high levels of internal consistency, with coefficients ranging from 0.75 to 0.96. The following eight items measuring child depressed affect, anxiety, hyperactivity, and self-regulation are used in this study: Apathetic/Withdrawn/Depressed Mood, Sober/Serious Mood, Anxious/Tense/Fearful Mood, Attentional abilities, Hyperactivity, Persistence, Impulsivity, and Self-regulation.

Revised Infant Behaviour Questionnaire (IBQ-R: [50]). The IBQ-R includes fourteen sub-scales: approach, vocal reactivity, high intensity pleasure, smiling and laughter, activity level, perceptual sensitivity, sadness, distress to limitations, fear, falling reactivity/rate of recovery from distress, low intensity pleasure, cuddliness, duration of orienting, and soothability. The sub-scales have adequate internal consistency (alpha = 0.71 to 0.90) and positive inter-rater agreement.

Ages & Stages Questionnaires (ASQ-3, ASQ-SE: [32], [33]). The ASQ provides an early, parent-reported evaluation of developmental progress. ASQ-3 sub-scales have good to acceptable internal consistency (alpha = 0.51 to 0.87), strong test-retest reliability (r = .75 to 0.82), and robust inter-rater reliability (r = .43 to 0.69). ASQ-SE sub-scales have adequate internal consistency (Cronbach’s alpha ranged from 0.67 to 0.91). ASQ-SE has test-retest reliability of 94%. Based on the results of our pilot study we chose to focus in this study on the problem solving domain of the ASQ-3 and the communication and self-regulation domains of the ASQ-SE at the 3- and 12-month postnatal time point. At the 24-month time point participants are asked to complete the communication, gross motor, fine motor, and problem solving ASQ-3 domains.

### Maternal measures

Structured Clinical Interview for DSM-5 – Clinician Version (SCID-5-CV: [44]). Diagnosis of current depressive disorder as an inclusion criterion is determined by a blinded psychologist via this gold-standard psychiatric interview conducted over the telephone. This interview is also used to determine other diagnoses or symptoms set as exclusion criteria (i.e., psychotic symptoms, bipolar disorder and substance use disorder). The tool has excellent inter-rater and test-retest reliability (kappa > 0.70) [51].

Beck Depression Inventory Revised (BDI-II: [52]) is a widely used, well-validated, 21-item clinical measure of severity of depression. The BDI-II has been validated against gold-standard diagnostic criteria in perinatal populations [53].

Beck Anxiety Inventory (BAI: [54]) is a 21-item measure of anxiety with well-established properties, including in perinatal populations [53].

Perceived Stress Scale (PSS: [55]) is the most widely used psychological instrument for measuring the perception of stress. This scale has acceptable internal consistency (alpha > 0.70) and test-retest reliability (r > .70) [56].

Parenting Stress Index (PSI: [57]) is a validated and well-researched 101-item parent report measure of parent-child relationship functioning and attachment.

Demographic and descriptive data. Data are collected on sample demographics (socio-economic indicators and pregnancy information; e.g., prenatal care, smoking, alcohol use, sleep patterns, history of depression during pregnancy), obstetric and birth information (e.g., birth weight, gestation, major complications), psychosocial factors (e.g., history of mental illness) and resource use (other services/medication accessed between follow-ups). Data on the impact of COVID-19 on maternal mental health, child behavioural development and related issues have been collected between January 2021 and February 2023.

### Safety monitoring

Maternal mood is monitored through questionnaires at four follow-up time points and also through the SCID at two of these time points. When risk of self-harm is indicated in the BDI-II [54] by either a positive score on item 9 and/or overall score within the severe range, or during the SCID, a risk assessment is completed. Participants deemed to be at low-to-moderate risk are referred to their GP and are provided with information on other available supports. Participants deemed to be at high risk are referred to receive immediate crisis attention [45].

### Data management

All participants are allocated a confidential trial code. The master coding database is kept in a secure computer drive with access restricted to the lead investigators, trial co-ordinator, and treating psychologist. All de-identified data are entered in a secure computer drive. Data entry is performed by the trial co-ordinator and data checks are performed regularly by the lead investigators. De-identified paper files are stored in locked filing cabinets, accessible only to the research team. Audio recordings of interviews and video recordings of parent-infant interaction are de-identified and are saved in secure computer drives only accessible to the research team. Only collated group data will be presented or published.

### Statistical methods

#### Power & Sample size calculations

The study is powered with respect to the primary outcome, the Internalising scale of the CBCL at 24 months. In our pilot data the control group standard deviation (SD) for the Internalising scale was 6.6. For the Internalising scale at 24 months, a between-group (treated vs. non-treated) mean difference of 3.3 points is considered the MCID as it equates to ½ SD difference [58, 59]. With SD = 6.6, the required n to detect a mean difference of 3.3 points in the Internalising scale with 80% power at alpha (two-tailed) = 0.05 is 15.7*(6.6/3.3)*2 = 63 per group. We typically see less than 20% loss to follow-up in our larger perinatal RCTs [48, 60]. For caution, we are allowing for a prudent margin of 25% attrition. This yields n* = 63/(1–0.25)2 = 112 which rounds to 115 per group (n = 230 in total).

A sensitivity analysis in G*Power confirms that with n = 230, both moderate and large effects have high reliability of being detected (power > 90%) and small effects will be reliably detected with power = 70%; even in models including up to six covariates. Thus, the study is sufficiently powered to detect the MCID in the primary child outcome (Internalising scale at 24 months).

#### Statistical analyses

Consistent with CONSORT standards [41], the primary analysis will be by intention-to-treat and will be conducted blind to treatment allocation. Baseline data will be secured prior to treatment allocation, and primary analyses will be executed twice: once using observed data, and once using multiple imputation, provided the assumptions for imputation are met [61]. The primary outcome (CBCL Internalising scale) will be analysed first using a 2-sample t-test comparing the intervention and control groups, extended to analysis of covariance (ANCOVA) to control for variation in baseline values. Potentially informative covariates, such as maternal antenatal anxiety, and potential mediators, such as maternal postnatal depression, will also be explored using a general linear model. The impact of the intervention on secondary outcomes, including the Externalising scale of the CBCL, and cognitive and motor development will also be explored using t-tests and ANCOVA. This analytical approach provides a direct and statistically sensitive test of the primary aim. In addition to inferential testing, effect sizes and their associated confidence intervals will be calculated. Finally, univariate logistic regression will be executed to determine any prognostic baseline variables that predict the return or non-return of follow-up data.

## Discussion

Maternal depression in pregnancy is highly prevalent and can have negative consequences on child emotional and behavioural development. Therefore, effectively treating depression in pregnant women can potentially prevent adverse emotional and behavioural development outcomes in their children. Our previous studies have demonstrated the effectiveness of our CBT intervention at reducing maternal depressive and anxiety symptoms during pregnancy [22] and showed promising results with respect to amelioration of child outcomes in our previous pilot study [22] and follow-up study [23]. The current RCT is adequately powered to detect the MCID in child internalising behaviour at 24 months following CBT intervention in pregnancy. CBCL internalising scores in early childhood are known to correlate with later mental health disorders [36, 37].

Susceptibility to emotional and behavioural problems in children of antenatally depressed mothers is thought to be exerted by antenatal and postnatal mechanisms as postulated by the model of Goodman and Gotlib [62]. The antenatal mechanism thought to mediate this susceptibility is dysfunctional neuroregulation due to fetal programming. This is supported by substantial evidence demonstrating the contribution of antenatal depression-induced alterations in the in-utero environment in predisposing the child to risk of psychopathology e.g., through changes in the hypothalamic–pituitary–adrenal axis function [17, 18] and other biological systems. Another biological mechanism underpinning this susceptibility suggested in the model is genetic predisposition. However, whilst the offspring’s DNA sequence is permanent, neuroregulation is shaped by the in-utero environment and could therefore conceivably be altered by antenatal psychological interventions (for instance if the intervention had a downstream effect on biological systems involved in programming e.g., cortisol levels [63]).

If our hypothesis that treating maternal depression in pregnancy can prevent adverse child outcomes is confirmed, this will contribute to our understanding of the impact of the in-utero environment on child outcomes and will have significant repercussions for standard antenatal care. In addition, this will better inform the delivery of maternal and infant health services and research into the prevention of the intergenerational transmission of mental health disorders.

Potential limitations: Given this is an RCT it is not possible to control for possible confounding influences that happen after randomisation, including postnatal influences such as maternal depression severity.

### Dissemination

The trial will be reported according to the CONSORT Statement for Randomised Trials of Nonpharmacologic Treatments [41]. Main findings will be submitted to a peer-reviewed journal for publication. Authorship will be determined by following the NHMRC Authorship Guidelines.

### Trial status and summary

This is an Australian-based randomised trial comparing the emotional and behavioural development of children whose mothers received an 8-week psychological treatment for antenatal depression to children whose mothers received treatment as usual. The protocol has been amended three times as detailed below and the current protocol version is 4 dated 5 August 2021. The trial was registered and approved on the Australian and New Zealand Clinical Trials Registry prospectively in 27 November 2018 and last updated 1 July 2022. Recruitment commenced at site 1 on 29 November 2018, at site 2 on 10 June 2020, at site 3 on 13 September 2021 and at site 4 on 2 November 2022. At the time of manuscript submission, 707 have been referred (self-referred 673, through hospitals 34) to the study, 177 have had a clinical assessment and 102 participants had been randomised to the trial (CBT n = 51; TAU n = 51).

### CONSERVE-SPIRIT extension statement

The trial was designed and commenced prior to the COVID-19 pandemic and a small number of modifications have been made to allow its continuation throughout the pandemic. Important modifications made in 2020 and 2021 (see online supplemental appendix 1) were: (1) delivery of the CBT intervention via telehealth; (2) distributing questionnaires via an online platform; (3) addition of COVID-19-related data items, such as how the pandemic affected the participant’s and her child’s mental health, to the questionnaires at all time points; (4) extension of the parent-reported ASQ-3 to the 24-month time point to allow data collection of children’s cognitive and motor development as a substitute to the in-person clinician-administered Bayley’s assessment; (5) extension of recruitment base by adding several maternity hospitals and opening the trial to women across Australia via the Ovia Pregnancy application; (6) extension of planned recruitment time frames to allow for slow recruitment due to government and/or local hospital COVID-19 restrictions and reorganisation of teams at recruiting sites; (7) resourcing additional funding to accommodate extended time frames. All modifications were made by the lead investigator Prof. Jeannette Milgrom. Modifications 3–5 which required protocol amendments were reviewed and approved by the ethics committee. Modifications 6–7 were reviewed and approved by funding bodies.

### Electronic supplementary material

Below is the link to the electronic supplementary material.

Supplementary Material 1

Supplementary Material 2

Supplementary Material 3

## Data Availability

The datasets used and analysed during the current study are available from the corresponding author on reasonable request.

## Abbreviations

ASQ-3: Ages & Stages Questionnaire
ASQ-SE: ASQ-Socio-Emotional Questionnaire
CBCL: Child Behaviour Checklist
CBT: Cognitive Behavioural Therapy
EPDS: Edinburgh Postnatal Depression Scale
GP: General Practitioner
MCID: Minimum Clinically Important Difference
PR: Post-Randomisation
RCT: Randomised Controlled Trial
IBQ-R: Revised Infant Behaviour Questionnaire
SCID-5-CV: Structured Clinical Interview for the Diagnostic and Statistical Manual of Mental Disorders-5 Clinical version-CV
TAU: Treatment As Usual
