# Supplementary material for: Early intervention to prevent adverse child emotional and behavioural development following maternal depression in pregnancy: study protocol for a randomised controlled trial
Source: BMC Psychol. 2023 Aug 4;11:222. doi: 10.1186/s40359-023-01244-w (PMC10401817; doi:10.1186/s40359-023-01244-w)
Supplement: Supplementary file 2 — Supplementary Material 2 [file 40359_2023_1244_MOESM2_ESM.pdf]

Supplement 2
for
Guidelines for reporting trial protocols and completed trials modified due to the COVID-19 pandemic and other extenuating circumstances: The CONSERVE 2020 Statement

CONSERVE Checklists

Use CONSERVE-CONSORT for completed trial reports and CONSERVE-SPIRIT for trial protocols.

**CONSERVE-CONSORT Extension: [DATE]**					
**Item**	**Item Title**	**Description**			**Page No.**
I.	Extenuating Circumstances	Describe the circumstances and how they constitute extenuating circumstances.			
II.	Important Modifications	a. Describe how the modifications are important modifications.			
		b. Describe the impacts and mitigating strategies, including their rationale and implications for the trial.			(see below)
		c. Provide a modification timeline.			
III.	Responsible Parties	State who planned, reviewed and approved the modifications.			
IV.	Interim data	If modifications were informed by trial data, describe how the interim data were used, including whether they were examined by study group, and whether the individuals reviewing the data were blinded to the treatment allocation.			
**CONSORT Number and Item**		For each row, if important modifications occurred check "direct impact" and/or "mitigating strategy" and describe the changes in the trial manuscript or supplement. Check "no change" for items that are unaffected in the extenuating circumstance.			**Page No.**
		**No Change**	**Impact***	**Mitigating Strategy****	
1	Title and abstract				
2	Introduction				
3	Methods: Trial Design				
4	Methods: Participants				
5	Methods: Interventions				
6	Methods: Outcomes				
7	Methods: Sample Size				
8-10	Methods: Randomisation				

11	Methods: Blinding				
12	Methods: Statistical methods				
13	Results: Participant flow				
14	Results: Recruitment				
15	Results: Baseline data				
16	Results: Numbers analysed				
17	Results: Outcomes and estimation				
18	Results: Ancillary analyses				
19	Results: Harms				
20	Discussion: Limitations				
21	Discussion: Generalisability				
22	Other information: Registration				
23	Other information: Protocol				
24	Other information: Funding				

*Aspects of the trial that are directly affected or changed by the extenuating circumstance and are not under the control of investigators, sponsor or funder.
**Aspects of the trial that are modified by the study investigators, sponsor or funder to respond to the extenuating circumstance or manage the direct impacts on the trial.

The CONSERVE-CONSORT Checklist is licensed by the CONSERVE Group under the Creative Commons Attribution-NonCommercial-NoDerivs 4.0 International license.

CONSERVE-SPIRIT Extension: [DATE] 20/12/2022

**Item**	**Item Title**	**Description**	**Page No.**
I.	Extenuating Circumstances	Describe the circumstances and how they constitute extenuating circumstances.	
II.	Important Modifications	a. Describe how the modifications are important modifications.	
		b. Describe the impacts and mitigating strategies, including their rationale and implications for the trial.	(see below)
		c. Provide a modification timeline.	
III.	Responsible Parties	State who planned, reviewed and approved the modifications.	
IV.	Interim data	If modifications were informed by trial data, describe how the interim data were used, including whether they were examined by study group, and whether the individuals reviewing the data were blinded to the treatment allocation.	

**SPIRIT Item and Number**		For each row, if important modifications occurred, check one or both of "impact" and/or "mitigating strategy" and describe the changes in the protocol. Check "no change" for items that are unaffected in the extenuating circumstance.			**Page No.**
		**No Change**	**Impact***	**Mitigating Strategy****	
1	Title	X			
2	Trial registration	X			
3	Protocol version	Minor changes	Updated (V4) - see Item 25 for details		
4	Funding		Extension to funding timelines due to COVID-19-related recruitment delays	Resourcing grant funds to allow trial completion	
5	Roles and responsibilities	X			
6	Background and rationale	X			
7	Objectives	X			
8	Trial design	X			
9	Study setting	X			
10	Eligibility criteria	X			
11	Interventions		Face-to-face delivery of intervention not conducted due to COVID-19 restrictions	Telehealth delivery of the CBT intervention	
12	Outcomes		Clinician-administered assess-ment of child's cognitive and motor development (secondary outcomes) not completed due to COVID-19 restrictions. COVID-19 might have an impact on the mental health of participants and their children in both groups	Addition of a parent-report tool for cognitive and motor development to the 24-month time point and COVID-19-related questions to all time points	

13	Participant timeline	X			
14	Sample size	X			
15	Recruitment		Slow recruitment due to COVID-19 restrictions and impact on recruiting teams at hospital sites	Establishing recruitment pathways through additiona maternity hospitals; extending recruitment Australia-wide through social media and extending recruitment time frame	
16	Allocation	X			
17	Blinding (masking)	X			
18	Data collection methods		Questionnaires cannot be distributed via mail due to preference of minimal contact	Questionnaires distributed via an online platform	
19	Data management	X			
20	Statistical methods	X			
21	Data monitoring	X			
22	Harms	X			
23	Auditing	X			
24	Research ethics approval	X			
25	Protocol amendments	Minor changes	Protocol was updated (V4) to reflect addition of COVID-19 related questions to the questionnaires at all time points, addition of parent-reported tool for the child's cognitive and motor development at the 24-month time point and addition of recruitment sites.		
26	Consent or assent	X			
27	Confidentiality	X			
28	Declaration of interests	X			
29	Access to data	X			
30	Ancillary and post-trial care	X			
31	Dissemination policy	X			
32	Informed consent materials	Minor changes	Patient information sheet and consent form were updated to reflect the addition of COVID-19 related questions		
33	Biological specimens	X			

*Aspects of the trial that are directly affected or changed by the extenuating circumstance and are not under the control of investigators, sponsor or funder.
**Aspects of the trial that are modified by the study investigators, sponsor or funder to respond to the extenuating circumstance or manage the direct impacts on the trial.

The CONSERVE-SPIRIT Checklist is licensed by the CONSERVE Group under the Creative Commons Attribution-NonCommercial-NoDerivs 4.0 International license.
